# Clinical manifestations of head and neck cancer in pediatric patients, an analysis of 253 cases in a single Brazilian center

**DOI:** 10.4317/medoral.25255

**Published:** 2022-04-03

**Authors:** Lady Paola Aristizabal Arboleda, Maria Eduarda Pérez-de-Oliveira, Iva Loureiro Hoffmann, Izilda Aparecida Cardinalli, Karen Patricia Dominguez Gallagher, Alan Roger Santos-Silva, Regina Maria Holanda de Mendonça

**Affiliations:** 1 Oral Diagnosis Department, Piracicaba Dental School, University of Campinas, Brazil; 2 Boldrini Children’s Center, Campinas, Brazil

## Abstract

**Background:**

Pediatric head and neck cancer (PHNC) is rare and its nonspecific clinical manifestations may often lead to delayed diagnosis. We aimed to describe the signs, symptoms, and clinicopathological characteristics of PHNC.

**Material and Methods:**

Medical records were retrospectively reviewed for all PHNC cases diagnosed from 1986 to 2016 affecting patients aged 19-years and younger from a tertiary referral center in Brazil. Demographic variables, anatomical site of primary tumors, histopathological diagnoses, signs and symptoms, and patterns of misdiagnosis were collected and interpreted by statistical and descriptive analysis.

**Results:**

A total of 253 PHNC cases were included. The mean age was 9.3 years and male patients were more frequently affected (60.9%). Burkitt lymphoma (23.7%), nasopharyngeal carcinoma (15.8%), and rhabdomyosarcoma (15.4%) were the most common cancer types. The nasopharynx (28.9%), cervical/lymph node region (25.3%), and craniofacial bones (8.3%) were the predominant anatomical sites. Tumor/swelling (68.4%), was the clinical finding often presented. The univariable analysis showed association between tumor histology and clinical variables such as sex (*p*=0.022), age (*p*<0.0001), anatomical location (*p*<0.0001) tumor/swelling (*p*=0.034), pain (*p*=0.031), systemic/general manifestations (*p*=0.004), nasal/breathing alterations (*p*=0.012), orbital/ocular alterations (*p*<0.0001). Misdiagnosis such as tonsillitis, otitis, and abscess were frequent.

**Conclusions:**

Although the clinical findings of PHNC are often unspecific, this study provided signs and symptoms with significant correlations between tumor histology. The suspicion of malignancy should be considered when the main signs and symptoms reported here appear and persist, in order to conduct a timely diagnosis.

** Key words:**Head and neck, cancer, children, adolescent, signs, symptoms.

## Introduction

Among several different cancer categories identified in pediatric patients, head and neck cancer (HNC) accounts for 2–15% of all childhood cases. Although uncommon, cancer remains one of the leading causes of death among children and adolescents around the world (1,2). Malignancies such as non-Hodgkin lymphoma (NHL), Hodgkin lymphoma (HL), rhabdomyosarcoma (RMS), nasopharyngeal carcinoma (NPC), thyroid carcinoma (TC), neuroblastoma, and salivary gland carcinoma represent the main group of pediatric head and neck cancer (PHNC) (3-8).

The clinicopathological profile of HNC between pediatric and adult patients is completely different, since squamous cell carcinoma (SCC) is the most common histopathological type in adults, with well-defined signs and symptoms suggestive of malignancy. In addition, there are a group of eleven mucosal disorders that may precede the diagnosis of oral SCC (9). However, the diagnosis of PHNC presents a challenging scenario due to the following particularities: the wide heterogeneity of cancer types; the nonspecific clinical findings for each cancer subtype; signs and symptoms similar to congenital, reactive and benign lesions, the most common head and neck pathologies in pediatric patients (10-12).

It is well recognized that pediatric head and neck masses, lymphadenopathy, fever, and other systemic signs and symptoms, are common clinical findings present in benign or infectious pathologies (13). However, the literature has shown some signs and symptoms more relevant than others (pallor, lump mass swelling head and neck, lymphadenopathy, abnormal movement, bruising, fatigue, bleeding, headache, visual impairment, pain, musculoskeletal symptoms) as they increase the probability of a cancer diagnosis, principally when children presented multiple times within 3 months (14). Despite this, most studies are focused on the most prevalent types of cancer in pediatric patients, such as leukemia, central nervous system (CNS) tumors, and lymphomas (1,15).

In order to assess the main signs and symptoms of PHNC, this study describes the clinical manifestations according to the most common histopathological subtypes in pediatric patients diagnosed with head and neck cancer in a tertiary referral center in Brazil for Pediatric Oncology and Hematology. The anatomical region of head and neck is evaluated by a multidisciplinary team, such as general practitioners, pediatric oncologist, pediatricians, otorhinolaryngologists, dentists, among others. Thus, the results of this study should be of interest to health professionals in order to raise awareness about the PHNC, and to favor a timely diagnosis when the main clinical-demographic characteristics of these tumors are present.

## Material and Methods

The retrospective study included pediatric patients less than 19 years of age diagnosed with primary HNC in the period from 1986–2016 at a tertiary referral center in Brazil. Medical records were reviewed for information regarding age, gender, race, anatomical site, histopathological diagnosis, signs and symptoms, and misdiagnosis. Clinical manifestations were collected according to the symptoms noted by the patient and the abnormal physical findings observed by a physician in the initial medical records.

Patients with CNS malignancies, retinoblastomas, HL, second primary tumors, metastases affecting the head and neck region were excluded due to most of those tumors belonging to specific medical fields (neurology, ophthalmology, hematology, among others). Furthermore, some malignancies may have their first clinical manifestation in different anatomies than the head and neck region or present generalized disease. Patients with incomplete medical records were also excluded. An age cut-off of 19 years was used to define the pediatric group and were distributed in two categories: ≤ 10 years and > 10 years. In addition, patients’ ethnicities were classified as white, black and other.

All tumors were classified using the International Classification of Diseases for Oncology ICD-O-3 and grouped according to the 4th edition of the WHO Classification of Head and Neck Tumors. The following categories were considered: nasal cavity; paranasal sinuses; nasopharynx; parapharyngeal space; oral cavity (tongue, gum, buccal mucosa, external upper lip, and palate); oropharynx (base of tongue, tonsils, adenoids); cervical/lymph node region; salivary glands; craniofacial bones; the ear; skin; thyroid gland; and orbit (7,16,17).

Signs and symptoms were classified into the following categories: tumor/swelling, cervical lymphadenopathy, pain, specific systemic manifestations, nasal/breathing alterations, oral and oropharyngeal alterations, orbital/ocular alterations, ear/hearing alterations, speaking alterations, and others (18). The count of signs and symptoms between each clinical manifestation group was considered through presence and absence in each of the 253 patients.

Data were collected in a datasheet, systematically organized in Microsoft Office Excel 2013 software (Microsoft Corporation, Redmond, WA, USA) and further analyzed by descriptive statistics using absolute numbers, percentages, mean values, and standard deviations. Posteriorly, analysis using SPSS software (IBM Corporation, Armonk, NY), version 22 was performed. The existence of associations between clinical variables and histological subtypes was assessed using the Pearson chi-square test or Fisher's test. For all tests, a 5% significance level was used.

## Results

A total of 7,181 malignant tumors were diagnosed in patients under 19 years of age, from 1986–2016 (30 years); of these, 5,724 (79.71%) were not primary tumors located in the head and neck region, 929 (12.94%) cases were CNS tumors, 251 (3.50%) were retinoblastomas, and 24 (0.33%) patients had incomplete medical records. The remaining 253 (3.52%) patients with malignant tumors were included in the study.

PHNC showed predominance of white male patients (60.9%) with a mean age of 9.3 years (±4.2 years), with sex (*p*=0.022) and age (*p*<0.0001) statistically significant when associated with tumor histology. Lymphomas (39.9%) were the most common cancer type followed by carcinomas (28.1%), and sarcomas (26.1%). The most common subtypes were BL (23.7%), NPC (15.8%), and RMS (15.4%). Table 1 provides the frequency of 253 PHNC cases by sex, mean age and ethnicity. Regarding anatomical location, the nasopharynx (28.9%) was the main anatomical site affected by PHNC, followed by cervical/lymph node region (25.3%), craniofacial bones (8.3%), and thyroid gland (8.3%). The statistical analysis showed that anatomical location (*p*<0.0001) was associated with tumor histology. Table 2 shows the association analysis between the clinical variables and tumor histology of PHNC, as well as the frequency and distribution of anatomical sites in relation to the main types of cancer.

**Figure 1:**
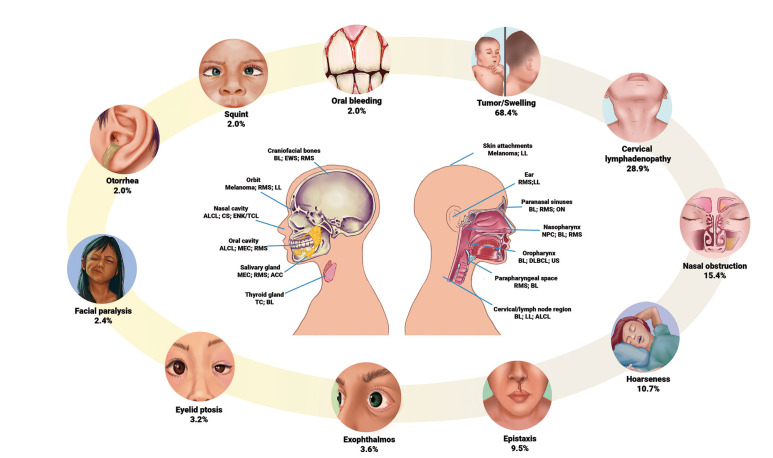
The most frequent anatomical sites and histopathological subtypes with the main clinical manifestations in PHNC.

According to clinical manifestations in PHNC, univariable analyses showed that tumor/swelling (*p*=0.034), specific systemic manifestations (*p*=0.004), nasal/breathing alterations (*p*=0.012), pain (*p*=0.031), and orbital/ocular alterations (*p*<0.0001) were related to the tumor histology (Table 3). Fig. 1 displays an overview of the main clinicopathological results, showing some common clinical manifestations of PHNC and the most frequent histopathological subtypes for each anatomical site. Table 4 shows a descriptive analysis related to signs and symptoms in PHNC by each histopathological type.

In addition to primary care physicians and pediatricians involved in the initial care of these patients, other health professionals, whose area of expertise is focused on the head and neck region, such as otorhinolaryngologists, dentists, and ophthalmologists, performed the first appointment and subsequently referred them to a pediatric oncology hospital for suspected malignancy in 19.6%, 8.8%, and 5.9% of the cases, respectively.

Information on the tentative primary diagnosis was available in 118 (46.6%) cases. Of these, sixty-one patients (51.7%) patients were suspected of having malignant disease. Infections such as tonsillitis, otitis, or abscess were the primary differential diagnosis in 42 cases (35.6%). Inflammatory lesions, cysts, and other benign diseases were the first diagnostic hypotheses in 15 (12.7%) cases.

**Table 1:**
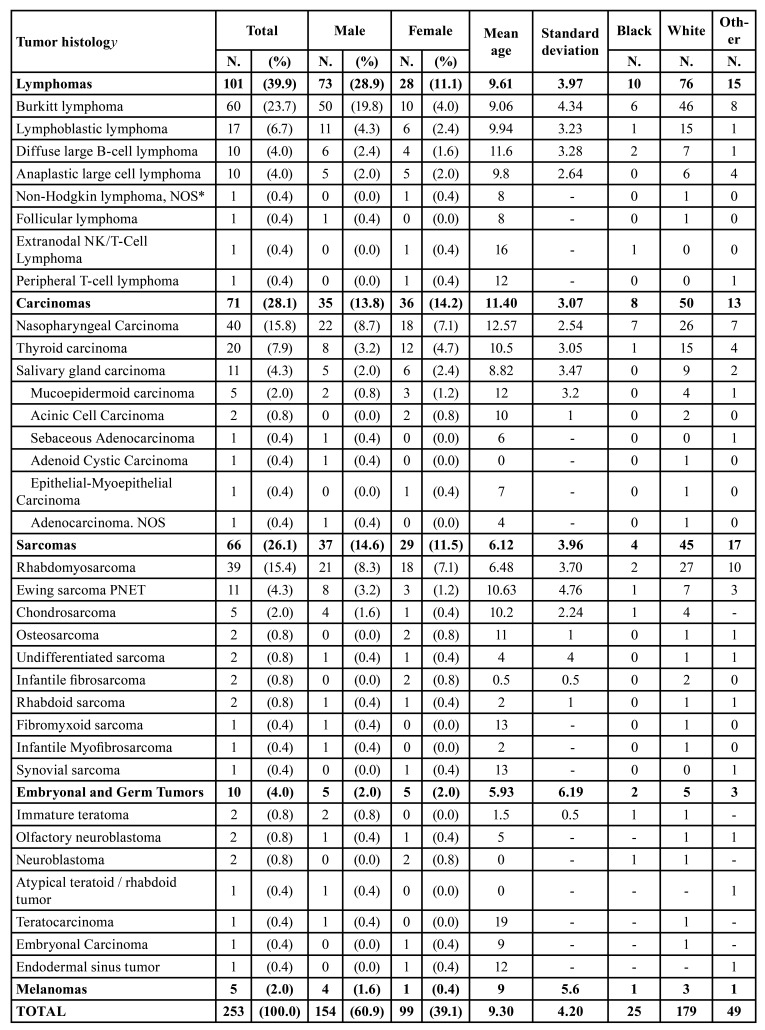
Frequency of PHNC by sex, mean age and race.

**Table 2:**
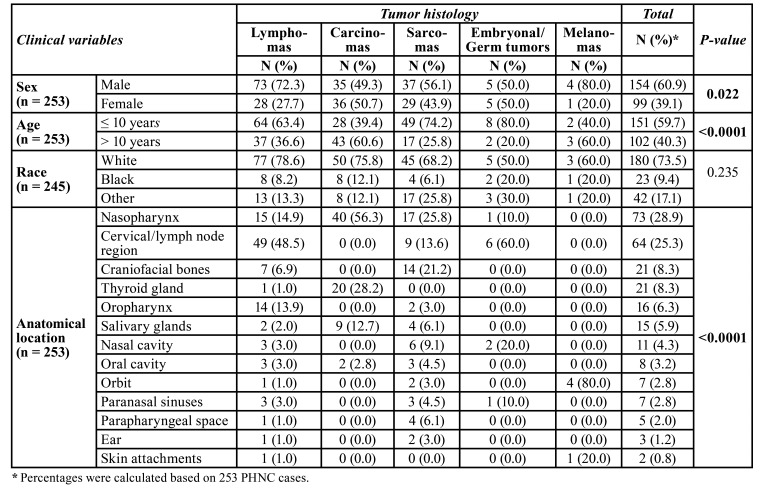
Association analysis between the clinical variables and tumor histology of the head and neck cancer in pediatric patients.

**Table 3:**
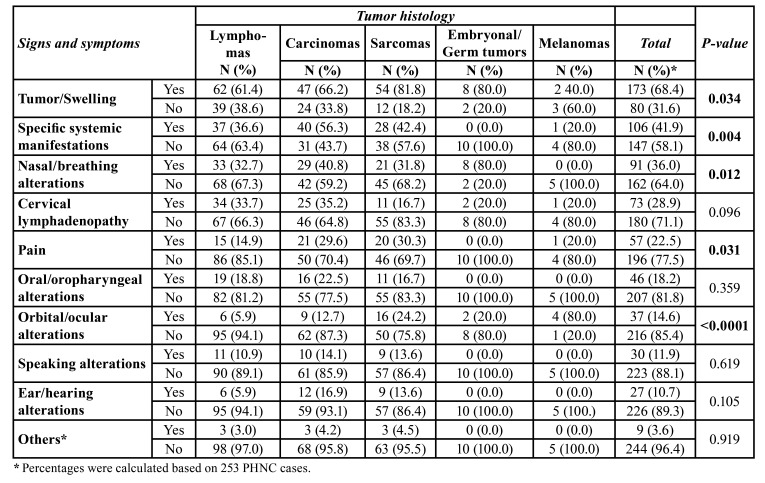
Association analysis between the signs and symptoms and tumor histology of the head and neck cancer in pediatric patients.

**Table 4:**
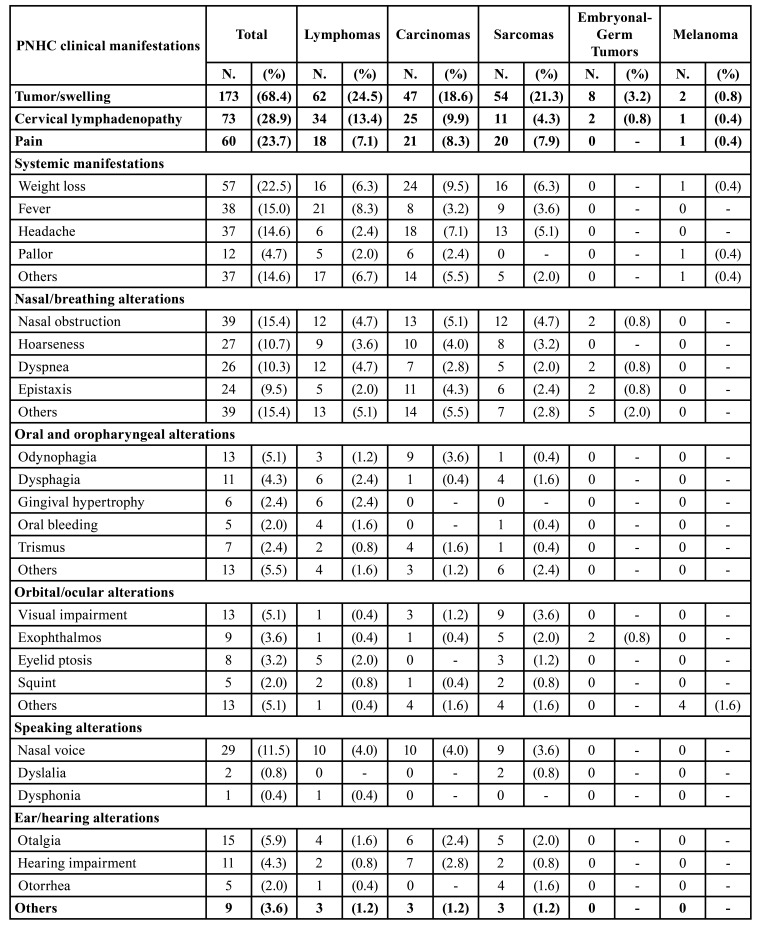
Main signs and symptoms in PHNC by each histopathological type.

## Discussion

The present study is consistent with the low incidence (3.52%) of PHNC that has been demonstrated worldwide (2). However, there are myriad malignant tumor subtypes that may affect this complex anatomical region. The early diagnosis of PHNC is particularly challenging because of the nonspecific clinical manifestations; in addition, the signs and symptoms are similar to other benign frequent pathologies in this population. Throughout this study, we provide an overview of the main signs and symptoms of PHNC in relation to the different patterns of cancer that affect the head and neck region in a large cohort of Brazilian pediatric patients.

The demographic characteristics of the PHNC agree with the findings of other studies. Commonly, there is a slight predominance of male patients and the average age at diagnosis varies between 7-10 years (12,19). However, some demographic particularities can be found in specific histopathological subtypes, for example, male patients with a higher prevalence of BL and female patients with predisposition to NPC (3). According to age groups, our results are similar to those previously reported. Thus, patients under 4 years of age have a higher frequency of germ cell tumors, embryonal tumors, and sarcomas. Patients aged 5-9 years are more prone to lymphomas, and patients aged 10-19 years are more likely to be affected by carcinomas (4,6,7).

PHNC distribution is in accordance with data obtained worldwide. Although the frequency varies depending on each geographic region, lymphomas, carcinomas, and sarcomas are the most common histological origin affecting the head and neck region in children and adolescents (2). Statistical analysis showed that the anatomical sites are directly related to the main histological subtypes, for example, the nasopharynx may be more affected by malignancies such as NPC, BL, and RMS; and lymphomas are more common in the cervical/lymph node region. These results are in agreement with those obtained in USA, Denmark, and Germany, with PHNC predominantly affecting anatomical locations such as nasopharynx, cervical/lymph node region, craniofacial bones, and thyroid gland, due to the high frequency of lymphomas, NPC, RMS, and TC (3,4,6).

Overall, the signs and symptoms reported in this study are in line with those of previous studies, with a tumor/swelling being the most common clinical finding in all cancer types (11). As mentioned in the literature, head and neck masses in pediatric patients are extremely common, and they often represent a clinical manifestation of congenital, inflammatory, and benign lesions (10). Thyroglossal cyst (31%), plunging ranula (17%), and lymphangioma (16%) have been the main diagnoses of head and neck masses in children and adolescents (20). However, it is important to emphasize that rapidly growing masses causing local destruction at presentation are features that should lead to greater suspicion of malignancy (12,21).

Specific systemic manifestations represent a group of relevant signs and symptoms, as they are usually identified in childhood cancer. Our study showed a significant association (*p*=0.004) among this group of findings and tumoral histology, with high frequency of signs and symptoms such as weight loss, fever, headache, and pallor. Although most studies have reported a high correlation of these symptoms in leukemias, we found a variety of results distributed in each histological origin, for example, fever was common in lymphomas, weight loss was frequently found in carcinomas, particularly NPC, and the headache was a common symptom in sarcomas (11,15). Dommett *et al*. (2013) identified symptoms that could highlight the possibility of cancer, among them, pallor was statistically significant for hematologic cancer. The authors concluded that a repeat attendance in a pediatric patient with pallor or a tumor/swelling in the head and neck region increased the risk of cancer to 0.76% (14).

Although cervical lymphadenopathy was another relevant and frequent clinical manifestation in this study, it was not statistically significant. However, we found a high frequency of this clinical feature in lymphomas and carcinomas, specifically NPC. Interestingly, the literature described this correlation due to the lymph nodes represented the main site affected by lymphomas, and the high frequency of cervical metastases caused by NPC (10). These results support the previous study that reported lymphadenopathy as a red flag for early recognition of malignancies such as lymphomas and NPC (15). Generalized or localized lymphadenopathy should be carefully evaluated when it is persistent or in combination with other findings, especially with the presence of B symptoms (fever, malaise, weight loss, and night sweats) as they suggest a possible malignancy (13). Clinical features of lymphadenopathy such as painless lymph nodes larger than 2-3 cm in diameter with hard, irregular and firm consistency have been reported as suggestive of malignancy (22). However, a recent study suggested that these features are not only associated with malignant tumors, since they observed also similar clinical features in 25–41% of chronic nonspecific lymphadenopathies and mycobacteriosis, and in 60% of acute/subacute lymphadenopathies (10).

The nasal cavity and nasopharynx were anatomical locations frequently affected in this study, and as expected, nasal/breathing alterations were statistically significant (*p*=0.012). These anatomical sites were affected mainly by tumors such as NPC, BL, RMS, Ewing sarcoma, Diffuse large B-cell lymphoma, and chondrosarcoma. The main clinical manifestations were nasal obstruction, snoring, dyspnea, epistaxis, and rhinorrhea. In accordance with the present results, it has been shown that manifestations such as nasal obstruction, epistaxis, chronic otorrhea, and dysphagia may be signs of NPC or other tumors located adjacent to this anatomical region (15). A retrospective cohort analysis of nasal cavity cancer in US pediatric patients found a prevalence of RMS and esthesioneuroblastoma. The signs and symptoms reported in this study seem to be consistent with our results, as clinical manifestations such as nasal obstruction, ophthalmological manifestations (proptosis, diplopia, lid discoloration, and loss of vision), epistaxis, headache, weight loss, lethargy, obstructive sleep apnea, anosmia, foul nasal discharge, and cervical lymphadenopathy were also commonly found in tumors affecting the nasal cavity (12). These results suggest that they are important manifestations to be evaluated, and imaging tests are indicated for the assessment of tumor masses affecting the nasal cavity, paranasal sinuses and nasopharynx.

The results obtained from the oral and oropharyngeal group were not statistically significant, however, signs and symptoms such as odynophagia, dysphagia, gingival hypertrophy, oral bleeding, and trismus, were found in the present study. The most prevalent tumors found in the oral and oropharyngeal regions were BL and RMS. Results from a systematic review that evaluated the clinical manifestations of lymphomas located in the palatine tonsils of pediatric patients were different to our findings. The authors found a prevalence of BL and the main signs and symptoms were unilateral tonsillar enlargement, alteration in appearance of the tonsil, and cervical lymphadenopathy (19). Although our study reported few clinical manifestations in the oral and maxillofacial region, it is important to emphasize clinical features such as bone destruction, tooth mobility, ulceration, tooth displacement, and oral bleeding, since they are frequent clinical signs of BL (21). Other clinical aspects of relevance for the dentist, and which were not reported in this study due to the exclusion criteria, are swelling and gingival bleeding, as these signs need to be carefully evaluated in order to exclude gingival infiltration of leukemia or Langerhans cell histiocytosis (15).

Regarding orbital/ocular alterations, the *p-value* was statistically significant (<0.0001) in this study, showing correlation among the clinical findings and the histological tumor. Speaking alterations, and ear/hearing alterations were less frequent, and no correlation was demonstrated. Noteworthy, descriptive analysis showed that signs and symptoms such as nasal voice, otalgia, hearing impairment, and visual impairment were related with NPC. According to Benoit *et al*. (2008), a possible explanation for the presence of these clinical manifestations might be that tumors located in the nasal region tend to be positioned more posterolaterally in the anterior cranial fossa, invading the ocular structures (12).

Regarding PHNC misdiagnosis, it is important to emphasize that due to the retrospective nature of the study, a limiting factor was obtaining this information in all evaluated cases. However, the types of primary differential diagnoses corresponded to those reported in the literature, with infection, inflammation, benign injury, and cyst being the main primary misdiagnosis (11). The high frequency of initial hypotheses of malignancy may be due to our study was performed in a tertiary referral center for Pediatric Oncology and Hematology, and most patients are already referred with a high suspicion of a malignant diagnosis.

Delay in the diagnosis of PHNC was not the aim of the present study, however, interesting results from two studies about this, showed the correlation between different signs and symptoms and delayed diagnosis. Benoit *et al*. (2008) studied nasal cancer in the pediatric population and demonstrated that the delay in diagnosis depended on the types of signs and symptoms, thus, patients who presented nonspecific complaints such as nasal obstruction, headache and fatigue, were diagnosed later (74 weeks) than those who presented focal manifestations such as proptosis, vision loss, epistaxis and anosmia (14 weeks) (12). Lilja-Fischer *et al*. (2019) demonstrated that general symptoms as fever, weight loss, pallor, fatigue and sweating were relevant for early diagnosis (41 days) compared with patients without general symptoms, who had much longer diagnostic intervals (34 - 120 days) (11). These results are very interesting, as they reflect the importance of a careful history and physical examination to discover all the signs and symptoms even if it were nonspecific.

Clinical information obtained from anamnesis and physical examination, represent the most important tools to generate an adequate diagnostic hypothesis and guide the professional in choosing the appropriate laboratory and radiological tests. Radiography, ultrasound, computed tomography, and magnetic resonance imaging are the most commonly used imaging methods to aid the clinician in the differential diagnosis of head and neck masses. Fine-needle aspiration diagnostic testing has also been shown to provide critical diagnostic information and avoid the need for open biopsy (13). The set of clinical, imaging and laboratory information can generate a high suspicion of malignancy, however, none of this information alone can make the final diagnosis. Thus, a surgical biopsy is mandatory for the definitive pathological diagnosis of head and neck lesions that are persistent and resistant to initial treatment (10,12).

The limitations of this study are based on its retrospective methodology, and the fact that the cases were collected in a hospital focused on the diagnosis and treatment of malignant tumors in pediatric patients. Consequently, these cases are referred with high suspicion of malignancy to physicians specialized in the oncological field. In addition, no information was available on cases referred with suspected HNC and which, after investigation, revealed pathologies of benign origin. Incomplete clinical information or difficult to interpret due to handwritten medical records were not analyzed, and some clinical aspects related to diagnostic hypotheses, or to the areas of health professionals who referred the patient, generated only descriptive results.

The diagnosis of childhood cancer is challenging, mainly for head and neck tumors, which is an anatomical region commonly affected by congenital or reactive lesions. Clinical manifestations associated with PHNC may be extremely vague and it often requires professional knowledge and experience to recognize warning signs and symptoms. The contribution of this study was to describe the signs and symptoms reported in the PHNC; thus, the identification of these clinical characteristics, especially tumor/swelling, specific systemic manifestations, and nasal/breathing alterations, should be carefully evaluated by the multidisciplinary team, in order to generate a high index of suspicion for carrying out diagnostic tests and timely referrals.

## References

[1] Siegel RL, Miller KD, Fuchs HE, Jemal A (2021). Cancer Statistics, 2021. CA Cancer J Clin.

[2] Arboleda LPA, de Mendonça RMH, Lopez EEM, Araújo ALD, Palmier NR, de Pauli Paglioni M (2020). Global frequency and distribution of head and neck cancer in pediatrics, a systematic review. Crit Rev Oncol Hematol.

[3] Albright JT, Topham AK, Reilly JS (2002). Pediatric head and neck malignancies: US incidence and trends over 2 decades. Arch Otolaryngol Head Neck Surg.

[4] Gosepath J, Spix C, Talebloo B, Blettner M, Mann WJ (2007). Incidence of childhood cancer of the head and neck in Germany. Ann Oncol.

[5] Schwartz I, Hughes C, Brigger MT (2015). Pediatric head and neck malignancies: incidence and trends, 1973-2010. Otolaryngol Head Neck Surg.

[6] Grønhøj C, Hjalgrim L, Jakobsen KK, Charabi B, Mirian C, Laier GH (2018). Incidence of head and neck cancer in children: A Danish nationwide study from 1978 to 2014. Pediatr Blood Cancer.

[7] Arboleda LP, Hoffmann IL, Cardinalli IA, Santos-Silva AR, de Mendonça RM (2018). Demographic and clinicopathological distribution of head and neck malignant tumors in pediatric patients from a Brazilian population: a retrospective study. J Oral Pathol Med.

[8] Benoit C, Orbach D, Cyrille S, Belhous K, Minard-Colin V, Kadlub N (2021). Head and neck tumors in children and adolescents: Impact of a multidisciplinary tumor board. Oral Oncol.

[9] Warnakulasuriya S, Kujan O, Aguirre-Urizar JM, Bagan JV, González-Moles MÁ, Kerr AR (2021). Oral potentially malignant disorders: A consensus report from an international seminar on nomenclature and classification, convened by the WHO Collaborating Centre for Oral Cancer. Oral Dis.

[10] Riva G, Sensini M, Peradotto F, Scolfaro C, Di Rosa G, Tavormina P (2019). Pediatric neck masses: how clinical and radiological features can drive diagnosis. Eur J Pediatr.

[11] Lilja-Fischer JK, Schrøder H, Nielsen VE (2019). Pediatric malignancies presenting in the head and neck. Int J Pediatr Otorhinolaryngol.

[12] Benoit MM, Bhattacharyya N, Faquin W, Cunningham M (2008). Cancer of the nasal cavity in the pediatric population. Pediatrics.

[13] Meier JD, Grimmer JF (2014). Evaluation and management of neck masses in children. Am Fam Physician.

[14] Dommett RM, Redaniel T, Stevens MC, Martin RM, Hamilton W (2013). Risk of childhood cancer with symptoms in primary care: a population-based case-control study. Br J Gen Pract.

[15] Fragkandrea I, Nixon JA, Panagopoulou P (2013). Signs and symptoms of childhood cancer: a guide for early recognition. Am Fam Physician.

[16] Person L, Lacour B, Faure L, Guissou S, Poulalhon C, Orbach D (2021). Childhood head and neck cancer in France: Incidence, survival and trends from 2000 to 2015. Int J Pediatr Otorhinolaryngol.

[17] Arboleda LPA, Hoffmann IL, Cardinalli IA, Gallagher KPD, Santos-Silva AR, Mendonça RMH (2020). Oral and maxillofacial cancer in pediatric patients: 30 years experience from a Brazilian reference center. Int J Pediatr Otorhinolaryngol.

[18] Hong X, Khalife S, Bouhabel S, Bernard C, Daniel SJ, Manoukian JJ (2019). Rhinologic manifestations of Burkitt Lymphoma in a pediatric population: Case series and systematic review. Int J Pediatr Otorhinolaryngol.

[19] Guimarães AC, de Carvalho GM, Bento LR, Correa C, Gusmão RJ (2014). Clinical manifestations in children with tonsillar lymphoma: A systematic review. Crit Rev Oncol Hematol.

[20] Xia X, Liu Y, Wang L, Xing Z, Yang L, Xie F (2019). Neck masses in children: a 10-year single-centre experience in Northwest China. Br J Oral Maxillofac Surg.

[21] Arboleda LPA, Rodrigues-Fernandes CI, Mariz BALA, de Andrade BAB, Abrahão AC, Agostini M (2021). Burkitt lymphoma of the head and neck: An international collaborative study. J Oral Pathol Med.

[22] Miranda Galvis M, Reis LAD, Santos-Silva AR, Vargas PA, Corrêa MB, Lopes MA (2017). Hodgkin lymphoma diagnosed during dental treatment: the importance of neck palpation. Gen Dent.

